# Exploring the Overlooked Depression, Anxiety, Insomnia and Fibromyalgia Syndrome Burden in Arab Women with Type 2 Diabetes: New Avenues for Chronic Disease Management

**DOI:** 10.3390/medicina60081304

**Published:** 2024-08-12

**Authors:** Omar Gammoh, Abdelrahim Alqudah, Maysa Alswidan, Lamia Abu Shwiemeh, Hanan Abu Shaikh, Talal Massad, Sereene Al-Jabari, Abdel-Ellah Al-Shudifat, Jafar Alsheyyab, Ammena Y. Binsaleh, Sireen Abdul Rahim Shilbayeh, Alaa A. A. Aljabali

**Affiliations:** 1 Department of Clinical Pharmacy and Pharmacy Practice, Faculty of Pharmacy, Yarmouk University, Irbid 21163, Jordan; 2019507282@alumni.yu.edu.jo (M.A.); 2019507058@ses.yu.edu.jo (L.A.S.); 2 Department of Clinical Pharmacy and Pharmacy Practice, Faculty of Pharmaceutical Sciences, The Hashemite University, Zarqa 13133, Jordan; abdelrahim@hu.edu.jo; 3 Prince Hamza Hospital, Amman 11947, Jordan; hananabushaikh@gmail.com; 4 Faculty of Medicine, The University of Jordan, Amman 11942, Jordan; massad.talal@gmail.com; 5 Faculty of Pharmacy, The University of Jordan, Amman 11942, Jordan; serenenedal2001@gmail.com; 6 Department of Medicine and Family Medicine, College of Medicine, The Hashemite University, Zarqa 13133, Jordan; abdel-ellah@hu.edu.jo; 7 Department of Internal Medicine, Faculty of Medicine, The Hashemite University, Zarqa 13133, Jordan; jafar@hu.edu.jo; 8 Department of Pharmacy Practice, College of Pharmacy, Princess Nourah bint Abdulrahman University, P.O. Box 84428, Riyadh 11671, Saudi Arabia; aysaleh@pnu.edu.sa (A.Y.B.); ssabdulrahim@pnu.edu.sa (S.A.R.S.); 9 Department of Pharmaceutics and Pharmaceutical Technology, Yarmouk University, Irbid 21163, Jordan; alaaj@yu.edu.jo

**Keywords:** insomnia, diabetes, women, side effects, fibromyalgia syndrome, depression, anxiety

## Abstract

*Background and Objectives*: Although physical health is always studied for women with diabetes, the mental health aspect is generally overlooked for this chronic disease. The present study aimed to examine the prevalence of psychosomatic symptoms, namely, fibromyalgia syndrome, depression, anxiety, and insomnia, and how these symptoms related to the medications used in a cohort of women diagnosed with type 2 diabetes (DM) in Jordan. *Materials and Methods*: This cross-sectional study recruited women diagnosed with type 2 diabetes, and validated scales (PSRS, PHQ-9, GAD-7, and ISI-A) for fibromyalgia syndrome, depression, anxiety, and insomnia were used. The associations between the different medications used and the dependent variables were examined using four separate multivariate logistic regression models. *Results*: Data were analyzed from 213 participants. Of them, 27.2% met the threshold for fibromyalgia syndrome diagnosis, 38% met the threshold for severe depression, 36.2% met the threshold for severe anxiety, and 39.9% met the threshold for severe insomnia. Fibromyalgia syndrome symptoms were significantly associated with glimepiride (OR = 1.92, CI = 1.00–3.68), β-blockers (OR = 2.21, CI = 1.03–4.70), diuretics (OR = 3.13, CI = 1.26–7.78), herbal remedies (OR = 2.12, CI = 0.98–4.55), and prescriptions for centrally acting medication (OR = 2.78, CI = 1.24–6.29). Significant associations were found between depression and diuretics (OR = 2.62, CI = 1.05–6.67), over-the-counter nonsteroidal anti-inflammatory drugs (NSAIDs) (OR = 3.49, CI = 1.69–7.23), and herbal remedies (OR = 5.07, CI = 2.40–10.69). In addition, anxiety was significantly related to diuretics (OR = 2.48, CI = 1.02–6.02), and OTC NSAIDs (OR = 2.60, CI = 1.29–5.21). Significant associations were evident between insomnia and β-blockers (OR = 3.23, CI = 1.51–6.95), acetaminophen (OR = 2.09, CI = 1.06–4.08), NSAIDs (OR = 4.61, CI = 2.18–9.76), and herbal remedies (OR = 5.95, CI = 2.71–13.07). *Conclusions*: Medications are associated with high burden of fibromyalgia syndrome, depression, anxiety, and insomnia. These findings underscore the importance of revising and optimizing the pharmacotherapy of these vulnerable patients, performing close mental health monitoring and the implementation of non-pharmacological interventions by integrating mental health services for women with chronic diseases such as diabetes.

## 1. Introduction

Diabetes mellitus (DM) is a global leading cardiovascular and metabolic disorder that is associated with macrovascular and microvascular complications [1]. Painful peripheral neuropathy and other musculoskeletal abnormalities have been reported in DM that impair the daily functioning of patients [2]. Chronic pain was reported in 60% of patients diagnosed with DM and is tightly related to psychological distress [3,4]. Fibromyalgia syndrome is a syndrome that highly prevails in women and is marked by its widespread pain, low mood, and sleep disturbance. The American College of Rheumatology (ACR) diagnostic criteria require finding at least 11 of 18 possible tender points on examination and unexplained symptoms persisting for at least three months [5]. Fibromyalgia syndrome prevails in approximately 5% of generally healthy populations [6]; however, this percentage spikes to 18–21% among patients diagnosed with DM, suggesting similar underlying pathology worth investigation [7,8]. Although the exact origin of fibromyalgia syndrome remains unclear, several physical and emotional stressors are implicated in this syndrome, including being female, genetic factors, and physical and mental trauma [9]. In addition, patients with diabetes exhibit alterations in inflammation and glucose metabolism that could contribute to fibromyalgia syndrome [10,11].

Mental health disturbances are comorbid with both diabetes and fibromyalgia syndrome [12,13]. The rates of depression associated with anxiety were shown to be higher in developing countries versus the developed ones. For example, according to a meta-analysis in one developing country, the prevalences of depression and anxiety were reported to be 46% and 32%, respectively, in patients with DM, compared to 32% for depression and 22% for anxiety in developed countries [14]. In addition, almost 40% of patients diagnosed with fibromyalgia syndrome were diagnosed with depression and anxiety disorders [15]. Furthermore, a diagnosis of fibromyalgia syndrome is associated with sleep problems in 60% to 80% of patients. This typically manifests as difficulties staying asleep, a problem that has been supported by polysomnography tests. Daytime drowsiness and weariness are other prominent and chronic fibromyalgia syndrome symptoms [16]. According to the literature, numerous studies have been dedicated to identifying the prevalence, incidence, risk factors, complications, and comorbidities of DM in the Jordanian population [17,18]; however, very few studies have been dedicated to examining the prevalence of fibromyalgia syndrome, depression, anxiety, and insomnia among women with type 2 diabetes. The identification of the prevalence and the correlates of fibromyalgia syndrome, depression, anxiety, and insomnia among patients with type 2 diabetes provides important insights for the proper psychiatric care for women to improve their daily functioning.

Therefore, the current investigation was undertaken to estimate the prevalence and to identify the correlates of fibromyalgia syndrome, depression, anxiety, and insomnia in in women diagnosed with type 2 diabetes (DM) in Jordan with a special focus on the long-term medication used. The authors hypothesize that the chronic medications used could affect the above-mentioned symptoms in this study sample.

## 2. Materials and Methods

### 2.1. Study Design and Settings

This cross-sectional study protocol gained approval from the IRB committee of Yarmouk University (175/2023). Women diagnosed with type 2 diabetes attending the internal medicine clinics at Prince Hamza Hospital in Amman and King Hussein Hospital in Irbid were approached.

### 2.2. Participants Recruitment

All information regarding the study objectives and steps were explicitly explained to the potential participants by the researchers (M.A and L.A). Later, on the same day, the study instrument was sent to the interested subjects. Before enrolling in the study, all the participants read the informed consent form and agreed electronically to enroll in the study. All participants had the freedom to pursue or to withdraw from participation. The sample size was decided based on previous literature where similar studies recruited 100–140 participants; however, in the current study, we aimed to recruit more than 200 women diagnosed with type 2 diabetes [7,19].

### 2.3. Study Instrument

A carefully designed study tool was developed to collect the demographic and clinical information of the study participants and to evaluate the four outcome variables.

### 2.4. Covariates

A self-administered well-structured online questionnaire comprising questions about the participant’s age, marital status, working status, highest received education, smoking status, and prior diagnosis with comorbidities was administered. In addition, the chronic medications used for diabetes and other chronic illnesses were recorded. Moreover, the over-the-counter analgesics used for fibromyalgia syndrome-like symptoms were recorded, such as acetaminophen “APAP”, non-steroidal anti-inflammatory drugs “NSAIDs”, herbal household remedies such as anise, or any prescription for centrally acting medication “CAM” for pain.

### 2.5. Outcome Measures

#### 2.5.1. Fibromyalgia Syndrome

The self-assessment of fibromyalgia syndrome was undertaken using the Patient Self-Report Survey (PSRS). The tool relies on the assessment of widespread pain, symptom severity, and the duration of symptoms for >3 months as per the diagnostic criteria of the ACR. A score ≥13 points with unexplained symptoms for 3 months was considered consistent with positive screening for fibromyalgia syndrome [20].

#### 2.5.2. Depression

The depressive symptoms were assessed through the translated and validated version of the Patient Health Questionnaire-9 (PHQ-9) [21]. This scale captures depression symptoms and their severity for the past 2 weeks and produces a score range between 0 and 27 with a cut-off threshold ≥15 indicating severe depression [22,23,24].

#### 2.5.3. Anxiety

The translated and validated General Anxiety Disorder-7 (GAD-7) was completed by the participants to assess anxiety severity. This tool comprises 7 elements that explore and measure anxiety severity for the last 2 weeks and yields a score ranging from 0 to 21 with a score of ≥15 pointing to severe anxiety [25,26,27].

#### 2.5.4. Insomnia

The screening for severe insomnia symptoms was undertaken with the help of the Insomnia Severity Index-Arabic version (ISI-A). The scale [28] consists of 7 questions with a score ranging between 0 and 28 with a cut-off score of ≥15 for severe insomnia symptoms [29].

### 2.6. Statistical Analysis

The demographic and clinical data of the participants were categorical; therefore, they were presented as frequencies and percentages. This study identifies four dependent variables, namely, fibromyalgia syndrome, depression, anxiety, and insomnia. The identification of the correlates of each of the dependent variables was performed through preliminary univariate binary logistic regression to inform the potential factors demonstrating *p* < 0.10. Subsequently, these factors were further fed into the multivariable models for each of the dependent variables as done in previous studies [30]. Statistical significance was set at two-sided *p <* 0.05, and estimates were set at 95% CI. Analysis was performed using SPSS (Version 23 by IBM, Armonk, NY, USA).

## 3. Results

### 3.1. Sample Characteristics

Data were analyzed from 213 patients: 152 (71.4%) were aged above 50 years, 151 (70.9%) were married, 105 (49.3%) had comorbid hypertension, 88 (41.3%) were on glimepiride, 70 (32.9%) were on insulin, and 68 (31.9%) were on ACEIs/ARBs. Regarding the analgesics utilized, 139 (65.3%) were on OTC APAP, 44 (20.7%) were on NSAIDs, 43 (20.2%) consumed homeopathy herbal remedies, 32 (15%) reported receiving a prescription for “CAM”, and 39 (18.3%) reported not using analgesics. The demographical and clinical data are shown in Table 1.

### 3.2. Prevalence of Fibromyalgia Syndrome, Depression, Anxiety, and Insomnia

A total of 58 (27.2%) of the participants reported a score above the threshold for fibromyalgia syndrome diagnosis. Severe depression was evident in 81 (38%) of the study sample participants. Severe anxiety was found in 77 (36.2%) of the sample, and severe insomnia prevailed in 85 (39.9%) of the study sample. The prevalences of fibromyalgia syndrome, depression, anxiety, and insomnia are shown in Table 2.

### 3.3. Correlates of Fibromyalgia Syndrome, Depression, Anxiety, and Insomnia

The multivariate model for fibromyalgia syndrome as the outcome variable was finally adjusted for “glimepiride”, “β-blockers”, “diuretics”, “herbal remedies”, and “Rx CAM” and revealed that fibromyalgia syndrome symptoms were significantly associated with “glimepiride” (OR = 1.92, 95% CI = 1.00–3.68, *p* = 0.04), “β-blockers”(OR = 2.21, 95% CI = 1.03–4.70, *p* = 0.04), “diuretics” (OR = 3.13, 95% CI = 1.26–7.78, *p* = 0.01), “herbal remedies” (OR = 2.12, 95% CI = 0.98–4.55, *p* = 0.05), and “Rx for CAM” (OR = 2.78, 95% CI = 1.24–6.29, 0.01).

The multivariate model for severe depression was finally adjusted for “diuretics”, “NSAIDs”, and “herbal remedies” and revealed that severe depression symptoms were significantly associated with “diuretics” (OR = 2.65, 95% CI = 1.05–6.67, *p* = 0.03), “OTC NSAIDs” (OR = 3.49, 95% CI = 1.69–7.23, *p* = 0.001), and “herbal remedies” (OR = 5.07, 95% CI = 2.40–10.69, *p* < 0.0001).

The multivariate model for severe anxiety was finally adjusted for “SGLT2-i”, “diuretics”, and “OTC NSAIDs” and revealed that severe anxiety symptoms were significantly associated with “SGLT2-I” (OR = 0.21, 95% CI = 0.04–0.96, *p* = 0.04), “diuretics” (OR = 2.48, 95% CI = 1.02–6.02, *p* = 0.04), and “OTC NSAIDs” (OR = 2.60, 95% CI = 1.29–5.21, *p* = 0.007).

The multivariate model for severe insomnia was finally adjusted for “β-blockers”, “OTC APAP”, “OTC NSAIDs”, and “herbal remedies” and revealed that severe insomnia was significantly associated with β-blockers (OR = 3.23, 95% CI = 1.51–6.95, *p* = 0.003), “OTC APAP” (OR = 2.09, 95% CI = 1.06–4.08, *p* = 0.03), “OTC NSAIDs” (OR = 4.61, 95% CI = 2.18–9.76, *p* < 0.001), and “herbal remedies” (OR = 5.95, 95% CI = 2.71–13.07, *p* < 0.001). The regression analysis results for all the dependent variables are presented in Table 3.

## 4. Discussion

The present study examined the prevalence of psychosomatic symptoms, namely, fibromyalgia syndrome, depression, anxiety, and insomnia, and how these symptoms are related to medication use in a cohort of women diagnosed with type 2 diabetes (DM) in Jordan. We report a high prevalence of fibromyalgia syndrome in comparison to existing literature; we also report high rates of depression, anxiety, and insomnia. Important risk factors were sulfonylurea, β-blockers, diuretics, NSAIDs, and herbal remedies.

This is, according to our knowledge, the first study carried out on a sample of Jordanian Arab women diagnosed with diabetes, and our results are similar to those from previous cross-sectional studies in another parts of the world [7,8], assuring that fibromyalgia syndrome prevalence in women with diabetes is higher compared to that in healthy women.

Diabetes complications include neuropathies and inflammation, and these hallmarks are also shared with fibromyalgia syndrome. It is estimated that more than half of adults with diabetes reported chronic pain. This may be explained by the higher pain sensitivity due to neuropathy/inflammatory status [3,31]. Another explanation for the high fibromyalgia syndrome prevalence is that the pain descriptors chosen by the patients for fibromyalgia syndrome and diabetic neuropathy are often similar, especially in the sensory symptoms; therefore, fibromyalgia syndrome could be positively screened in a higher percentage of the patients [32].

In addition, our findings from the women of Jordan revealed high rates of severe depression, anxiety, and insomnia compared to the healthy population. This is consistent with existing literature where patients with diabetes were found to report higher mental distress in comparison to healthy peers [13,14,32]. Depression and diabetes have a bi-directional relationship. Patients experiencing depression usually adopt negative behaviors such as lack of compliance with therapy, unhealthy eating habits, and lack of exercise, which exacerbate diabetes. On the other hand, diabetes, is a chronic disease and its complications manifest hormonal disturbances and inflammation, which could worsen depression [33,34].

In our study, fibromyalgia syndrome was associated with the use of sulfonylurea, β-blockers, and diuretics. Although no evidence exists that these medications could lead to or exacerbate fibromyalgia syndrome or chronic pain, this can be explained by the severity of diabetes and its comorbidities, as mentioned above. Almost 40% of the recruited sample reported using sulfonylurea, and about one-third reported using insulin, which means that patients are uncontrolled on metformin and lifestyle modifications alone. Despite the lack of information about the full patient profiles, a deviation from the recent guidelines is evident. For example, none of the patients reported using glucagon-like peptide (GLP-1) analogs or dipeptidyl peptidase inhibitors (DPP-4i), and very few patients were on SGLT2-is. This can be explained by the lack of availability of these medications due to financial constraints [35]. Therefore, the poor management of diabetes and its complications could also explain the relatively high prevalence of fibromyalgia syndrome and mental health disturbances [36,37].

The present study revealed that NSAID utilizers were at higher odds for fibromyalgia syndrome, depression, anxiety, and insomnia. The ACR guidelines recommend the use of SNRI and anti-seizure medications rather than over-the-counter NSAIDs, which could exacerbate symptoms. We suggest that, due to poor awareness among the patients and the health care providers, only patients experiencing severe pain symptoms rely on NSAIDs more than acetaminophen. Evidence showed disappointing results for NSAIDs in fibromyalgia syndrome, which could explain the association between NSAID use and the poor outcomes of fibromyalgia syndrome, depression, anxiety, and insomnia [38,39]. Moreover, the use of herbal remedies was associated with severe depression, fibromyalgia syndrome, and insomnia. The authors suggest that these women with high burdens of the above-mentioned symptoms tend to depend on household self-remedies with herbal products, which show significant antinociceptive effects [38,39].

The present study is the first to examine the prevalence and the correlates of fibromyalgia syndrome, depression, anxiety, and insomnia in a cohort of women diagnosed with DM in Jordan. The study idea, the sample type and size recruited, and the validated assessment scales are all strengths of the study. On the other hand, the lack of detailed and updated laboratory tests for the A1C% and the lack of clinical examination for fibromyalgia syndrome versus diabetic neuropathy for all the recruited patients are considered limitations of the study. Therefore, future studies should employ detailed and thorough clinical examination to diagnose fibromyalgia syndrome in addition to comprehensive laboratory results to evaluate the clinical condition of the patients.

## 5. Conclusions

In conclusion, Jordanian women with diabetes experience a high burden of insomnia, fibromyalgia syndrome, depression, and anxiety associated with their long-term medications and the add-on analgesics. These findings underscore the importance of revising and optimizing the pharmacotherapy of these vulnerable patients, performing close mental health monitoring, and the implementation of non-pharmacological interventions to alleviate their psychosomatic symptoms. This can be achieved by integrating mental health clinics with the outpatient clinics serving patients with chronic diseases.

## Figures and Tables

**Table 1:** Demographics and clinical details of the study sample (n = 213).

Factor	Category	n (%)
Age	<50 years	61 (28.6)
	≥50 years	152 (714)
Marital status	Single	62 (29.1)
	Married	151 (70.9)
Education level	High school	152 (71.4)
	University	61 (58.6)
Employment status	Unemployed	182 (85.4)
	Employed	31 (14.6)
Smoking status	Non-smoker	149 (70)
	Smoker	64 (30)
Comorbid hypertension	Yes	105 (49.3)
Chronic medications and OTC analgesics used:		
Glimepiride		88 (41.3)
Insulin		70 (32.9)
SGLT-2i		19 (8.9)
ACEIs/ARBs		68 (31.9)
β-blockers		35 (16.4)
Diuretics		24 (11.3)
OTC APAP		139 (65.3)
OTC NSAIDs		44 (20.7)
Homeopathy herbal remedies		43(20.2)
Rx for CAM		32 (15)
No analgesics		39 (18.3)

SGLT2-i: Sodium-glucose co-transporter 2 inhibitor; ACEIs/ARBs: angiotensin-converting enzyme inhibitors/angiotensin 2 receptor blockers; OTC: over-the-counter; APAP: acetaminophen; NSAIDs: non-steroidal anti-inflammatory drugs; CAM: centrally acting medication.

**Table 2:** The prevalences of fibromyalgia syndrome, depression, anxiety, and insomnia in a cohort of women diagnosed with diabetes (n = 213).

Outcome Variable	Category	n (%)
Fibromyalgia syndrome (Patient self-report survey)	Below threshold	155 (72.8)
	Above threshold	58 (27.2)
Severe depression (PHQ-9)	Below threshold	132 (62)
	Above threshold	81 (38)
Severe anxiety (GAD-7)	Below threshold	136 (63.8)
	Above threshold	77 (36.2)
Severe insomnia (ISI-A)	Below threshold	128 (60.1)
	Above threshold	85 (39.9)

**Table 3:** Regression analysis demonstrating the association between the dependent variables and the independent variables.

Fibromyalgia Syndrome						
	Univariate Analysis			Multivariate Analysis		
Factor	OR	95% CI	*p*	OR	95% CI	*p*
Age	0.85	0.44–1.64	0.63			
Marital status	0.99	0.51–1.92	0.97			
Education	0.64	0.32–1.30	0.22			
Employment	1.33	0.58–3.02	0.49			
Smoking	1.33	0.70–2.52	0.39			
Comorbid hypertension	2.49	1.33–4.68	0.004			
Glimepiride	1.97	1.07–3.64	0.02	1.92	1.00–3.68	0.04 *
Insulin	0.56	0.28–1.11	0.10			
SGLT2-i	0.69	0.22–2.18	0.53			
ACEIs/ARBs	1.78	0.95–3.30	0.07			
β-blockers	2.30	1.12–4.70	0.02	2.21	1.03–4.70	0.04 *
Diuretics	3.10	1.30–7.39	0.01	3.13	1.26–7.78	0.01 *
OTC APAP	1.57	0.81–3.04	0.18			
OTC NSAIDs	1.51	0.74–3.09	0.25			
Herbal remedies	1.80	0.88–3.67	0.10	2.12	0.98–4.55	0.05
Rx for CAM	3.31	1.52–7.18	0.002	2.78	1.24–6.29	0.01 *
No analgesics	0.33	0.12–0.90	0.03			
**Severe depression**						
	Univariate analysis			Multivariate analysis		
Factor	OR	95% CI	*p*	OR	95% CI	*p*
Age	0.63	0.35–1.15	0.13			
Marital status	0.59	0.33–1.09	0.09			
Education	0.98	0.53–1.81	0.95			
Employment	0.88	0.40–1.95	0.75			
Smoking	0.97	0.53–1.77	0.92			
Comorbid hypertension	1.18	0.68–2.05	0.56			
Glimepiride	0.96	0.55–1.68	0.89			
Insulin	1.13	0.63–2.03	0.67			
SGLT2-i	0.41	0.13–1.27	0.12			
ACEIs/ARBs	1.22	0.67–2.19	0.51			
β-blockers	1.45	0.73–2.91	0.29			
Diuretics	2.10	0.89–4.95	0.08	2.65	1.05–6.67	0.03 *
OTC APAP	1.45	0.80–2.62	0.22			
OTC NSAIDs	2.99	1.51–5.92	0.002	3.49	1.69–7.23	0.001 *
Herbal remedies	4.72	2.03–8.35	<0.0001	5.07	2.40–10.69	<0.0001 *
Rx for CAM	2.07	0.97–4.40	0.06			
No analgesics	0.30	0.12–0.7	0.006			
**Severe anxiety**						
	**Univariate analysis**			**Multivariate analysis**		
Factor	OR	95% CI	*p*	OR	95% CI	*p*
Age	0.83	0.45–1.52	0.54			
Marital status	0.71	0.39–1.29	0.26			
Education	0.81	0.43–1.52	0.52			
Employment	1.33	0.61–2.89	0.47			
Smoking	1.09	0.59–1.99	0.78			
Comorbid hypertension	1.01	0.57–1.75	0.99			
Glimepiride	1.67	0.95–2.95	0.07			
Insulin	1.53	0.85–2.75	0.15			
SGLT2-i	0.18	0.04–0.83	0.03	0.21	0.04–0.96	0.04 *
ACEIs/ARBs	1.14	0.63–2.07	0.67			
β-blockers	1.59	0.79–3.19	0.19			
Diuretics	2.80	1.17–6.65	0.02	2.48	1.02–6.02	0.04 *
OTC APAP	1.55	0.84–2.83	0.15			
OTC NSAIDs	2.62	1.34–5.16	0.005	2.60	1.29–5.21	0.007 *
Herbal remedies	1.52	0.77–3.01	0.22			
Rx for CAM	1.46	0.68–3.12	0.33			
No analgesics	0.64	0.30–1.38	0.25			
**Severe insomnia**						
	**Univariate analysis**			**Multivariate analysis**		
Factor	OR	95% CI	*p*	OR	95% CI	*p*
Age	0.59	0.32–1.07	0.08			
Marital status	0.74	0.41–1.34	0.31			
Education	0.80	0.43–1.47	0.47			
Employment	0.57	0.25–1.31	0.19			
Smoking	1.15	0.63–2.07	0.66			
Comorbid hypertension	1.18	0.68–2.04	0.56			
Glimepiride	0.99	0.57–1.73	0.97			
Insulin	1.43	0.80–2.55	0.23			
SGLT2-i	0.67	0.24–1.84	0.44			
ACEIs/ARBs	0.99	0.55–1.78	0.97			
β-blockers	2.19	1.08–4.42	0.03	3.23	1.51–6.95	0.003 *
Diuretics	1.59	0.67–3.72	0.29			
OTC APAP	1.78	0.99–3.23	0.05	2.09	1.06–4.08	0.03 *
OTC NSAIDs	3.43	1.72–6.87	<0.001	4.61	2.18–9.76	<0.001 *
Herbal remedies	4.2	2.06–8.60	<0.001	5.95	2.71–13.07	<0.001 *
Rx for CAM	1.88	0.88–4.01	0.10			
No analgesics	0.27	0.11–0.64	0.003			

SGLT2-i: sodium-glucose co-transporter 2 inhibitor; ACEIs/ARBs: angiotensin-converting enzyme inhibitors/ angiotensin 2 receptor blockers; OTC: over-the-counter; APAP: acetaminophen; NSAIDs: non-steroidal anti-inflammatory drugs; CAM: centrally acting medication; OR: odds ratio; CI: confidence interval. * *p* < 0.05.

## Data Availability

Data will be available from the corresponding author upon request.
